# Utilisation of the partogram among nurses and midwives in selected health facilities in the Eastern Province of Rwanda

**DOI:** 10.4102/curationis.v40i1.1751

**Published:** 2017-08-03

**Authors:** Oliva Bazirete, Nomafrench Mbombo, Oluyinka Adejumo

**Affiliations:** 1 Department of Midwifery, School of Nursing and Midwifery, College of Medicine and Health Sciences, University of Rwanda, Rwanda; 2 Department of Health, Western Cape Government, South Africa

## Abstract

**Background:**

Maternal mortality continues to be a global burden, with more than 200 million women becoming pregnant each year and a large number dying as a result of complications of pregnancy or childbirth. The World Health Organisation has recommended use of the partogram to monitor labour and delivery in order to improve healthcare and reduce maternal and foetal mortality rates.

**Objective:**

This study described factors affecting utilisation of the partogram among nurses and midwives in selected health facilities of Rwanda.

**Method:**

A descriptive quantitative and cross-sectional research design was used. The population comprised 131 nurses and midwives providing obstetric care in 15 health institutions (1 hospital and 14 health centres). Data collection was through a self-administered questionnaire, and a pre-test of the data collection instrument was carried out to enhance validity and reliability. The Statistical Package for Social Sciences (version 21) was used to capture and analyse data. Ethical clearance was obtained from the University of the Western Cape (Republic of South Africa) and from the Institutional Review Board of Kigali Health Institute (Rwanda). Patricia Benner’s model of nursing practice was used to guide the study.

**Results:**

It was found that 36.6% of nurses and midwives did not receive any in-service training on how to manage women in labour. Despite fair knowledge of the partogram among nurses and midwives in this study, only 41.22% reported having used the partogram properly, while 58.78% reported not having done so.

**Conclusion:**

Nurses’ and midwives’ years of professional experience and training in managing pregnant women in labour were found to be predictors of the likelihood of proper use of the partogram. In-service training of obstetric caregivers in the Eastern Province of Rwanda is recommended to improve use of the partogram while managing women in labour.

## Introduction

Maternal mortality continues to be a global burden, with at least 585 000 women dying each year from complications of pregnancy and childbirth (Kitila et al. 2014). There were an estimated 289 000 maternal deaths worldwide in 2013, a decline of 45% from 1990. Sub-Saharan Africa alone accounted for 62% (179 000) of global deaths, followed by southern Asia (24%; 69 000) (World Health Organisation [WHO] et al. 2014). The African Union Commission and United Nations Population Fund (2013) report that the lifetime risk of dying because of pregnancy- or childbirth-related complications for women in Africa is 1 in 39, while in developed countries the risk is 1 in 3800.

Global initiatives to strengthen policy intervention for maternal mortality started with the WHO’s Safe Motherhood Initiative in 1987 (Horton 2010). The aim was to raise awareness about the numbers of women dying each year from complications of pregnancy and childbirth, with a target to reduce maternal morbidity and mortality by 50% by the year 2000 (Magon 2011). According to Horton (2010), the initiative did not succeed, although maternal health has always been a major focus of the WHO’s efforts.

The United Nations states that by 2030 the global maternal mortality ratio is expected to be reduced to less than 70/100 000 live births and neonatal mortality to at least 12/1000 live births (Alkema et al. 2016) to achieve the Sustainable Development Goals 2030. Rwanda still has high maternal mortality and neonatal mortality rates at 210/100 000 and 20/1000 live births, respectively (National Institute of Statistics of Rwanda 2016). A nationwide facility-based retrospective cohort study of maternal death in Rwanda by Sayinzoga et al. (2016) found 70% of maternal deaths to be because of direct causes. Postpartum haemorrhage was shown to be the leading direct cause of maternal death (22.7%), followed by obstructed labour (12.3%), obstetric infection (10.3%) and eclampsia (9.4%) (National Institute of Statistics of Rwanda 2016; Sayinzoga et al. 2016).

According to Opiah et al. (2012), midwives form the bulk of the skilled birth attendants in all levels of healthcare. Their knowledge of monitoring of pregnant women in labour using the partogram is thus a significant factor for prevention of obstructed labour and other complications that may arise during labour, among others postpartum haemorrhage, obstructed labour and uterine rupture.

To avoid the risk of complications or maternal death, women should be assisted during delivery by personnel who have received training in normal childbirth and who are able to diagnose, treat, and refer complications if needed. Almost 91% of deliveries in Rwanda are assisted by a skilled health provider: 18% by medical doctors, 70% by nurses and medical assistants and only 3% by midwives (National Institute of Statistics of Rwanda 2016). This is partly because of the availability of nurses in health facilities and the limited number of medical doctors and midwives in Rwanda (National Institute of Statistics of Rwanda 2016).

Most maternal deaths and complications attributable to obstructed and prolonged labour could be prevented by cost-effective and affordable health interventions, like use of the partogram (Magon 2011). The partogram (sometimes known as the partograph) is usually a preprinted paper form on which labour observations are recorded. The aim of the partogram is to provide a pictorial overview of labour, in order to alert midwives and obstetricians to deviations in maternal or foetal well-being and labour progress (Lavender, Hart & Smyth 2008). According to Soni (2009), the WHO advocates use of the partogram as a necessary tool in the management of labour and recommends its universal use during obstetrical labour. The literature suggests utilisation of the partogram for close follow-up of expectant women during labour and the early postpartum period. The objective of the WHO’s recommendation is to improve healthcare and reduce maternal and foetal morbidity and death (Magon 2011).

A host of researchers declare that effective use of the partogram requires knowledge and skills. Yisma et al. (2013) ascertained that utilisation of the partogram was significantly higher among obstetric caregivers working in health centres (67.9%) compared to those working in hospitals (34.4%). The tool was appreciated as an effective and safe instrument to monitor expectant women during labour in Ghana; however, skilled birth attendants have not consistently ‘bought in’ to use of the partogram, and it was adequately completed in only 25.6% of cases (Gans-Lartey et al. 2013).

With regard to place of delivery in Rwanda, in the Eastern Province (predominantly rural) 88.9% of deliveries are assisted by skilled service providers, while in the urban areas this rises to 94.5% (National Institute of Statistics of Rwanda 2016). Owing to the shortage of skilled birth attendants in Rwanda, it is not rare that nurses and midwives attending pregnant women in labour encounter difficulties while using the partogram.

The Rwanda Ministry of Health (2016) in its reference manual of Emergency Obstetrics and Neonatal Care recommends using the partogram as it clearly differentiates normal from abnormal progress in labour and identifies timeously those women likely to require intervention. However, there is limited literature regarding nurses’ and midwives’ knowledge and use of the partogram in the health facilities of Rwanda. There is therefore a need to describe factors affecting utilisation of the partogram among nurses and midwives in Rwanda, focusing on the Eastern Province where human resources are insufficient to safely assist deliveries.

## Purpose of the study

The purpose of this study was to describe factors affecting the utilisation of the partogram among nurses and midwives in selected health facilities of Rwanda.

## Research questions

The following three research questions were central to this study: What is the knowledge and use of the partogram among nurses and midwives in maternity wards of selected health facilities? What are the challenges facing nurses and midwives with regard to utilisation of the partogram in labour wards of selected health facilities? What factors influence the use (proper or not) of the partogram by nurses and midwives in maternity wards in selected health facilities?

## Research method and design

### Study design

This study adopted a descriptive quantitative and cross-sectional research design to gather information on utilisation of the partogram among nurses and midwives in selected public health facilities. A quantitative design is useful when wanting to describe and examine relationships and determine causality among variables (Grove, Burns & Gray 2013).

### Setting

The present study was conducted in Rwanda, a country located in east central Africa and covering an area of 26 338 square kilometres. According to projections, Rwanda’s population would grow to 11 274 221 in 2015 (National Institute of Statistics of Rwanda 2015). The country has five provinces: North, South, East, West and Kigali City. The national language is Kinyarwanda, and the official foreign languages are French and English. The Eastern Province is the largest and most populous but least densely populated of Rwanda’s five provinces. It has seven administrative districts, and one of these represented the study site for this study.

The setting of the present study included 1 hospital with a catchment population of 267 525 and 14 health centres. The hospital receives the largest number of maternity cases, referred by the 14 public health centres in the catchment area. According to the annual report of Rwamagana Hospital (Rwamagana District Hospital 2012), this facility conducts nearly 350 deliveries per month. All these 15 health facilities were included in the present study.

### Sampling

The study population and sample included all nurses and midwives who provide obstetric care to pregnant women during labour in the 15 selected health facilities. Considering the shortage of qualified skilled birth attendants in the Rwanda health system (National Institute of Statistics of Rwanda 2016), both nurses and midwives working in health centres are still attending pregnant women in labour. According to Grove et al. (2013), the entire population may be the target of a study when the population is small and well defined. Hence, the study results could be generalised to nurses and midwives of selected health facilities in the Eastern Province where the study was conducted. Health professionals who do not attend labour cases in selected health facilities were excluded from participating in the study.

There was a total of 170 nurses and midwives working in the selected health facilities; however, only 140 were considered as part of the study population as 30 of them were not available. Some were on annual, maternity, or sick leave, while others were purely involved in administrative work or attending in-service training in another province. A total of 140 nurses and midwives participated in the study, and of the 140 questionnaires distributed, 1 questionnaire had missing data and 8 were not returned. Therefore, a total of 131 questionnaires were completed and analysed in the present study.

### Data collection

A self-administered questionnaire consisting of closed-ended questions was used to collect data. Permission to use and adapt the original questionnaire was granted by its author, Opiah et al. (2012). Adaptations were made to four sections of the questionnaire: demographic characteristics, knowledge of the partogram, characteristics of partogram utilisation and factors affecting utilisation of the partogram for labour monitoring. These adaptations were made to some items in these four sections of the original questionnaire to fit the local context of the study.

The dependent variable of this study is the use (proper/not proper) of the partogram by nurses and midwives in selected health facilities, while the independent variables are socio-demographic variables and other important variables under study. The questionnaire was translated from English into French by a professional translator; both languages are officially used in Rwanda. The data collection instrument was translated to facilitate respondents’ understanding of the tool by using the language with which they are most conversant. Translation from French back into English was done by an independent professional translator, to confirm that the meaning and content of the original copy had not been changed during translation. Verification of the translated instrument was also done to ensure its validity. The original instrument for data collection that was adapted to the present research had demonstrated a high degree of validity and reliability with a coefficient of 0.89 at an alpha level of 0.05 (Opiah et al. 2012). A pre-test of the data collection instrument was carried out on 10 nurses and midwives out of the study setting to enhance the validity and reliability of the modified questionnaire. These nurses and midwives were not included in the sample size of this study. A research assistant was trained to facilitate data collection. The data collection was conducted during the first two weeks in August 2013.

The researcher in collaboration with the hospital director and officers in charge of the health centres made an appointment to meet with nurses and midwives on day shift. The questionnaires were given to all nurses and midwives who were willing to participate in the study after having signed a consent letter. The investigator explained the purpose and nature of the study, as well as the matter of confidentiality, and they were requested not to include any names on the questionnaire. It was explained that completing the questionnaire may take 30 minutes of their available time during their day shift. They were requested to complete the questionnaire as soon as possible and to return it to the investigator or research assistant within two days.

### Data analysis

All 131 completed questionnaires were captured using Statistical Package for Social Sciences (SPSS) version 21. After data collection, the researcher proceeded with data entry, followed by data cleaning and data screening to ensure that there were no errors or missing data. Data were analysed in three steps: firstly, univariate analysis was used to summarise data in terms of frequency distributions of the variables under study. Secondly, bivariate analysis was used to determine the relationship between the dependent variable (use of the partogram by nurses and midwives in the Rwamagana health facilities) and independent variables (sociodemographic and others). In this case, cross-tabulations together with the chi-squared test for independence were employed for categorical variables. Lastly, multivariate analysis was conducted to find out the extent to which independent variables affect dependent variables, using the logistic regression model. Captured and analysed data were reviewed by a statistician at the Department of Statistics at the University of the Western Cape.

## Ethical consideration

In undertaking this research, various sources were consulted to ensure that the study adhered to acceptable ethical guidelines. Participants in this study gave their consent to take part after receiving explanations about the purpose of the study as detailed in the information sheet. The original instrument for data collection that was adapted to the present research had demonstrated a high degree of validity and reliability with a coefficient of 0.89 at an alpha level of 0.05 (Opiah et al. 2012). A pre-test of the data collection instrument was carried out to enhance the validity and reliability of the modified questionnaire. Ethical clearance was obtained from the University of the Western Cape (13/6/22) and Kigali Health Institute (KHI/IRB/25/2013) and permission from Rwamagana Hospital (No14/209/HOP/RGNA/2013). After obtaining ethical clearance and permission, the investigator started with pre-testing of the questionnaire prior to data collection. Pre-testing of the instrument took place with 10 nurses and midwives who were not included in the sample.

## Results

A total of 140 questionnaires were distributed among nurses and midwives attending to pregnant women in labour in the selected health facilities and 131 questionnaires were fully completed, giving a response rate of 93.5%. One of the questionnaires had missing data and was excluded from analysis, and a further eight were not returned. Hence, data from 131 (*N* = 131) self-administered questionnaires were analysed using SPSS version 21.

### Distribution of respondents by age, gender, professional qualification and years of experience

Of the 131 nurses and midwives, 58.8% were women and 41.2% were men. The participants’ ages ranged from 23 to 61 years, with a mean age of 33.7 years (SD = 6.9 years). The ages were put into six different groups with age intervals of 5 years. Most respondents were enrolled nurses (74.8%) with a secondary level of education. Only 9.9% of respondents were midwives. More than half of the participants (55.0%) had professional experience of 0–4 years, 28.2% had 5–9 years, 7.6% had 10–14 years and 9.2% had 15 years and above.

### Distribution of respondents by training received in management of labour, place of work and unit of service

It was found that more than half of the participants (63.4%) had received training in management of pregnant women in labour: 55.7% were trained in Emergency Obstetric and Neonatal Care (EmONC) and 7.6% were trained in Advanced Life Support in Obstetrics (ALSO). The remaining 36.6% of nurses and midwives declared that they had not received any in-service training on management of labour.

Of the participants, 89.3% affirmed that they had been trained in using the partogram. The source of knowledge of using the partogram was given as the college or teaching institution by 51.1% of participants, while 48.9% did not respond.

Only 19.8% were working in the hospital, while 80.2% were working in health centres; 51.1% of respondents were permanently working in labour wards. The remaining 48.1% participants were working in units affiliated to labour wards (antenatal clinic, family planning and postnatal wards). All nurses and midwives who participated in this study were exposed to pregnant women in labour. Even though some participants were not found in the labour wards during the time of data collection, they rotate in different services and attend to pregnant women in labour, especially during night shifts and over the weekends. Forty-five per cent of participants revealed that while working in labour wards the shift is made up of only one staff member; 33.6% said there are two staff per shift, 20.6% that there are three staff per shift and just 0.8% said that the shift is made up of four persons.

### Nurses and midwives’ knowledge of the partogram

The results show that all 131 (100.0%) nurses and midwives have used the partogram previously, and 80.9% of them agreed that it is a simple graphic recording of progress of labour and salient conditions of the mother and foetus against time in hours. Of the 131 respondents, 79.4% knew the partogram was one of the tools to implement the Safe Motherhood programme, while 20.6% disagreed. A large percentage (90.1%) acknowledged use of the partogram in reducing maternal mortality, with 87.8% agreeing that this tool plays a key role in reducing newborn mortality. Of the respondents, 58.0% agreed that the action line of the partogram plot falls on the left of the alert line, while 42.0% disagreed.

Less than half of the respondents (32.1%) agreed that the correct role of the action line on the partogram is to alert them to take appropriate action, while 67.9% disagreed. The majority of participants (78.6%) attributed the role of the action line to the wrong function – indicating that it allows time for the woman to be adequately assessed for appropriate intervention. Also, only 54.2% of respondents were of the view that in normal labour the minimum duration of a strong contraction is 40 seconds, while 45.8% opposed this view. Results further showed that 68.7% of respondents claimed that at least 10 minutes is required to assess adequacy of uterine contractions effectively.

### Characteristics of partogram utilisation and challenges

A large number of participants (92.4%) were of the view that it is a managerial policy that all women in labour should be monitored with a partogram in respective health facilities, whereas 3.82% disagreed and 3.82% revealed that they did not know. However, only 30 nurses and midwives (22.9%) considered the partogram useful in obstetric review, while 3.1% disagreed and 74.0% did not know. Most respondents failed to describe the two important lines on the partogram, namely the alert line and the action line. Only 58.0% of nurses and midwives agreed that in normal progress of labour the graph or plot on the partogram should fall on the left of the alert line, with 42.0% disagreeing. Of all participants, 32.1% agreed with the correct role of the action line, which is to indicate the appropriate action to be taken for a pregnant mother in labour.

In addition to there being insufficient numbers of staff to monitor pregnant women in labour, it was found that the partogram is misused by most of the respondents. Only 41.2% were found to use the partogram properly, whereas 58.8% did not do so. Proper use of the partogram refers, in the current study, to monitoring of the pregnant mother in labour and recording findings at least once every 30 minutes. Nurses and midwives start completing the partogram when the woman enters the active phase of labour. In this regard, 41.2% of nurses and midwives monitored pregnant women and recorded every 30 minutes, 16.8% recorded every hour, 23.7% every 4 hours, 9.9% every 6 hours and 8.4% recorded the pregnant mother’s information after 12 hours.

All respondents (100%, *N* = 131) agreed that the partogram is available in their health facility. Of the respondents, 93.9% agreed that there is a need to develop managerial guidelines or protocols for each maternity unit in their health facility to facilitate effective use of the partogram, while 6.1% disagreed. Almost all participants (93.9%) opted to be trained in the use of the partogram.

### Factors influencing proper or not proper use of the partogram

In order to identify some of the factors that affect the use of the partogram, the chi-squared test for independence was used. The level of significance was set to 5%, with any *p*-value less than 0.05 indicating a statistically significant association between the two variables. Correlations were computed between the dependent variable (proper or not proper use of the partogram) and participants’ demographic characteristics (age, professional qualification, number of staff members per shift, years of experience and place of work) and other selected variables under study (training received and knowledge of use of the partogram).

The results indicated that among the factors believed to affect the use of the partogram, those having a significant association are professional qualification, years of professional experience and in-service training received. Of the 13 (9.9%) registered midwives who responded in this study, 8.4% used the partogram properly, while 1.5% did not. Of 18 (13.7%) registered nurses, 8.4% used the partogram properly and 5.3% did not. The Pearson chi-squared test revealed a statistically significant association between proper/not proper use of the partogram and professional qualification of nurses and midwives (χ^2^_(3)_ = 17.414, *p* = 0.001). Furthermore, among those with professional experience of 0–4 years, out of 72 nurses and midwives only 16% used the partogram properly, while 38.9% did not. Data show a significant association between number of years of professional experience and proper/not proper use of the partogram by nurses and midwives (χ^2^_(3)_ = 10.547, *p* = 0.014).

With regard to in-service training received, of the nurses and midwives who received training in management of labour, 34.4% used the partogram properly while 29.0% did not. Among those trained in EmONC, 30.5% used the partogram properly while 25.2% did not. Among nurses and midwives trained in ALSO, 2.3% used the partogram properly and 5.3% did not. A statistically significant association was found between correct use of the partogram and training received in management of labour (χ^2^_(1)_ = 15.789; *p* = 0.000).

A statistical association was not found between use of the partogram and number of staff per shift or place of work. Of 59 nurses and midwives (45.0%) who revealed that there is one staff member per shift in the labour ward, 15.3% used the partogram properly, whereas 29.8% did not. There was also no relationship found between use of the partogram and the shortage of nurses and midwives attending to pregnant women in labour (χ^2^_(3)_ = 6.479, *p* = 0.091). The results did not show a difference in proportions.

Similarly, results from this study did not indicate a significant relationship between use of the partogram and place of work (i.e. between proper/not proper use of the partogram among nurses and midwives working in a hospital or in health centres). There was also no significant link between proper/not proper use of the partogram and knowing the role of the partogram in terms of reducing maternal and neonatal mortality.

### Logistic regression predicting likelihood of proper or not proper use of the partogram

Binary logistic regression was performed to assess the impact of a number of factors on the likelihood that nurses and midwives would report properly or not properly using the partogram. The model contained various independent variables, but only two were found to be significant: number of years of professional experience and being trained in the management of pregnant women in labour. The full model containing these predictors was statistically significant, as shown by the Hosmer–Lemeshow goodness-of-fit test, with a chi-square value of 5.923 and a significance level of 0.432 at a degree of freedom of 6. With the Hosmer-Lemeshow goodness-of-fit test to support the logistic model, the *p*-value is expected to be greater than 0.05 (Pallant 2011). Results of this test indicate that the model was able to distinguish between respondents who are more likely to use the partogram properly and those less likely to do so. Only two independent variables made a unique, statistically significant contribution to the model: training in management of pregnant women in labour (*p* = 0.000) and number of years of experience (*p* = 0.050). Place of work did not contribute significantly.

The strongest predictor of reporting proper use of the partogram was being trained in management of pregnant women in labour (odds ratio 4.847; 95% confidence interval [CI] 2.005–11.719). This indicated that nurses and midwives trained in management of pregnant women in labour were over four times more likely to use the partogram properly than those who did not have this training. On the other hand, the odds ratios of 0.310 and of 0.745 for nurses with 0–4 and 5–9 years of experience were less than 1.000, indicating that respondents in these categories were, respectively, 0.310 and 0.745 times less likely to use the partogram properly than nurses and midwives with more experience. Table 1 shows the likelihood of the proper use of the partogram found in this study.

**TABLE 1:** Logistic regression predicting the likelihood of proper or not proper use of the partogram.

Independent variables	*B*	Wald	dfs	*p*	Odds ratio	95% CI for odds ratio	
						Lower	Upper
**Training in management of labour†**							
Yes	1.578	12.282	1	0.000	4.847	2.005	11.719
No (*r*)	-	-	-	-	1.000	-	-
**Years of experience†**		**7.474**	**3**	**0.05**			
0–4 years	−1.171	2.888	1	0.89	0.310	0.08	1.197
5–9 years	−0.259	0.165	1	0.684	0.745	0.18	3.081
10–14 years	0.269	0.08	1	0.777	1.309	0.202	8.469
15 years or more (*r*)	-	-	-	-	1.000	-	-
**Place of work**							
Hospital	−0.174	0.121	1	0.728	0.841	0.816	2.238
Health centre (*r*)	-	-	-	-	1.000	-	-
Constant	−0.711	0.894	1	0.344	0.491	-	-

† Predicators with a significant *p*-value; *r*, reference category; *df*, degree of freedom; *B*, regression coefficients.

## Discussion

The primary aim of this study was to describe factors affecting use of the partogram among nurses and midwives in selected health facilities of Rwanda. The response rate of 93.5% implies that this study was needed in Rwanda. The discussion is organised according to the study’s specific objectives: knowledge and use of the partogram, challenges facing nurses and midwives during utilisation of the partogram and factors affecting utilisation of the partogram

### Knowledge and use of the partogram among nurses and midwives

The results indicate that 80.9% of all of respondents (131) knew what a partogram was and more than three-quarters (79.4%) knew its role as a tool to implement the Safe Motherhood programme. The data in terms of knowledge of nurses and midwives also confirm the results of Uwimana (2008), who evaluated use of the partogram in one of Rwanda’s urban hospitals and showed that most midwives and nurses (88%) reported that a partogram is a tool for decision-making in the labour ward, with 98% confirming that correct use of the partogram can improve management of pregnant women in a labour ward.

However, knowledge of the function of the alert line and the action line was poor; only 76 (58.0%) respondents could explain the function of the alert line and 42 (32.1%) the function of the action line. This may indicate the need for urgent steps to improve the knowledge of nurses and midwives on the partogram through training and seminars, in order to maximise proper use of this tool. This is confirmed by the findings of Yisma et al. (2013), where 53.3% of respondents could correctly explain the function of the alert line while 53 (27.2%) and 38 (19.5%) gave an incorrect explanation or did not know the correct function, respectively.

These results are, however, higher than those found in a study carried out in Nigeria (Fawole, Hunyimbo & Adekanle 2008), where about 16.6% of respondents could explain the function of the alert line and 24.3% could explain the function of the action line. This could be because of differences in the pre-service training of nurses and midwives in Rwanda and Nigeria. This implies that the knowledge of nurses and midwives on the partogram may be inadequate for better use of this tool in health institutions where the studies were conducted. This was also confirmed by studies conducted in Nigeria (Fawole et al. 2008; Opiah et al. 2012) and Ethiopia (Yisma et al. 2013).

The college or teaching institution was reported as the primary source of knowledge by most (51.1%) of those who received training on the partogram. The low percentage of midwives (9.9%) who responded in this study might be why so few respondents reported having received pre-service training on the partogram (from school or teaching institution). The source of knowledge about the partogram in this study is similar to the findings of a study conducted in Ethiopia by Yisma et al. (2013).

In addition to education received from school, of all nurses and midwives who participated in this study, 73 (55.7%) were trained in EmONC and 10 (7.6%) in ALSO, while 48 (36.6%) did not receive any in-service training in management of the pregnant woman in labour. Similarly, a life-saving skills training workshop was reported as the primary source of knowledge by one-third of those who were aware of the partogram in a study in Nigeria (Oladapo, Daniel & Olatunji 2006). This indicates that knowledge from pre-service training needs to be updated by planned on-the-job training for better monitoring of pregnant women using the partogram. The nurses’ and midwives’ knowledge of the partogram was generally reported to be fair among those in the current study, and this is supported by other recent studies (Opiah et al. 2012; Yisma et al. 2013).

Despite fair knowledge of the partogram, there was poor use of it in labour monitoring, considering that the WHO recommended its widespread use for all women during labour (Nyamtema et al. 2008). Less than half of the participants (41.2%) were found to be using the partogram properly, and 58.8% were not. Several similar studies in Africa confirm low use of the partogram (Fawole, Adekanle & Hunyinbo 2010; Fawole et al. 2008; Khonje 2012; Ogwang, Karyabakabo & Rutebemberwa 2009; Opiah et al. 2012; Yisma et al. 2013). Inadequate knowledge and improper use of this simplified tool could be part of the reason for the high maternal mortality in developing countries, especially in Africa (Opiah et al. 2012; Saviola, Sudha & Metgud 2009; Yisma et al. 2013). This highlights the need for regular pre-service and on-the-job training of nurses and midwives on use of the partogram to safely monitor pregnant women in labour.

### Challenges facing nurses and midwives during utilisation of the partogram

With respect to the professional qualifications of respondents in the present study, the findings confirm the problem of a shortage of skilled birth attendants in Rwanda. The National Institute of Statistics of Rwanda (2016) reports that 18% of deliveries are assisted by medical doctors, 70% by nurses or medical assistants and only 3% by midwives. This is partly because of the availability of nurses in health facilities and the limited number of medical doctors and midwives in Rwanda. In comparison, in the study conducted by Yisma et al. (2013), most of the respondents were midwives, while public health officers constituted just one-sixteenth of respondents.

All the nurses and midwives who participated in this study attended to pregnant women in labour, and of the 131 respondents, only 9.9% were midwives. The majority of respondents were enrolled nurses. According to Opiah et al. (2012), midwives form the bulk of the skilled birth attendants at all levels of healthcare; their knowledge in partographic labour monitoring is thus a significant factor in prevention of obstructed labour. Furthermore, the findings of this study reveal a shortage of staff per shift in labour wards. The majority of respondents (45.0%) claimed that when they worked, there was one person per shift, 33.6% that there were two, 20.6% that there were three and 0.8% that there were four per shift.

The present study suggests regular pre-service training of midwives and deployment of skilled birth attendants in rural health facilities. To buttress this, Fatusi et al. (2008), in a study to evaluate health workers’ training in use of the partogram, reported that lower cadres of primary healthcare workers can be effectively trained to use the partogram with satisfactory results, and thus contribute towards improved maternal outcomes in developing countries with a scarcity of skilled attendants. The majority of nurses and midwives who participated in this study reported the need to develop managerial guidelines or protocols on how to use the partogram to ensure that it was used properly.

The above suggestion aligns with the overall objective of Human Resources for Health (HRH) under the Ministry of Health of Rwanda, as stipulated by the third Health Sector Strategic Plan, which is to ensure availability of an adequate, equitably distributed, qualified, motivated and productive workforce responsive to the country’s changing needs and demands (Rwanda Ministry of Health 2012). One of its priorities is expanding and strengthening the capacity of teaching institutions to augment HRH production, the target being to reach midwife/population and nurse/population ratios of 1/25 000 and 1/1000, respectively, in 2018. The baseline midwife/population and nurse/population ratios in 2011 were 1/66 749 and 1/1294, respectively (Rwanda Ministry of Health 2012).

In contrast to previous studies (Ogwang et al. 2009; Opiah et al. 2012) reporting the problem of non-availability of the partogram in health facilities, all the participants in the present study reported that partograms were available in their health institutions.

### Factors influencing proper or not proper use of the partogram among nurses and midwives

Although all nurses and midwives interviewed in this study had formal training on how to use the partogram, the impact of such training was not reflected in their performance; the tool was poorly utilised, with only 41.2% reporting proper use. Results revealed a statistically significant association between use of the partogram and whether the nurses and midwives had received in-service training, number of years of experience, shortage of staff as well as professional qualification.

Results from the present study also revealed that there was a significant relationship between proper/not proper use of the partogram and whether the nurses and midwives had received in-service training (at a degree of freedom of 1, *p* = 0.000; a strong relationship). This is in line with other literature confirming this relationship (Nyamtema et al. 2008; Ogwang et al. 2009). Being trained in management of pregnant women in labour is also a strong predictor in the logistic model reporting proper use of the partogram, with an odds ratio of 4.847 (CI 2.005–11.719). This may be because of the programme of the Rwanda Ministry of Health (2012) to train skilled birth attendants countrywide in EmONC. These findings were confirmed by Yisma et al. (2013), who found that obstetric caregivers who had not previously been trained on the partogram had lower odds of using it, compared to those who had been trained.

Number of years of professional experience was another predictor of proper use of the partogram: the more years of professional experience nurses and midwives have in practice, the more likely they are to use the partogram properly. This is similar to findings from Opiah et al. (2012), who found support for the relationship between knowledge and years of professional experience. This also responds to the conceptual framework that guided the present study, which is the model of nursing practice put forth by Benner, Tanner and Chelsa (1996), which states that nurses and midwives develop and improve their nursing skills by exposure to and experience of real situations in the clinical field. In the present study, knowledge and clinical skills of nurses and midwives in using the partogram seem to improve as they pass through the competency levels of Benner et al.’s model, from novice to advanced beginner, then competent, proficient and expert.

The results of this study also reveal an association between the number of nurses and midwives per shift and proper use of the partogram or not. Shortage of staff was confirmed in the literature (Opiah et al. 2012) as a factor influencing utilisation of the partogram.

Ogwang et al. (2009) found use of the partogram to be regarded as an additional time-consuming task. In his study, some participants were of the opinion that the partogram was a detailed tool that was not practical to use when there is only one midwife in the unit, as they have to execute other duties such as administering treatment, managing labour, providing health education and offering family planning services, among others. This might be a reason for poor utilisation of the partogram in the present study, as most of the nurses and midwives confirmed that the shift in the labour ward is usually made up of only one or two staff members. Kinfu et al. (2009), in a study to estimate the inflow and outflow of health workers in Africa, suggest that pre-service training needs to be expanded and combined with other measures to increase health worker inflow. The aim is to reduce the rate of outflow to reach the WHO’s recent target of 2.28 professionals per 1000 population for countries taken as a whole (Kinfu et al. 2009).

Another factor found to influence proper use of the partogram was professional qualification. This again is closely linked to the shortage in the midwifery workforce in Rwanda (Rwanda Ministry of Health 2012); there are also not enough registered nurses to effectively attend to pregnant women in labour across the country. Saviola et al. (2009) confirm the relationship between professional qualification and use of the partogram. The present study suggests regular formal training combined with in-service training of nurses and midwives for better monitoring of pregnant women in labour.

## Limitation of the study

The study was only conducted in 15 health facilities in one province of Rwanda. With that consideration, the findings cannot be generalised to the whole country because it is limited to some health facilities in the Eastern Province. Moreover, the methodology, especially the quantitative design used in this study to describe factors affecting the utilisation of partogram, generated data that could not assess the quality in documenting the partogram and determine the outcomes of the mother and the newborn. To this end, there is a need for qualitative study to improve delivery of care to pregnant women in labour.

## Conclusion

For many years, the partogram has been used as a standard for monitoring labour, the main reason being the assumption that it would guide in early identification of problems during labour and hence assist in taking appropriate action that could lead to reduction in complications of pregnancy and birth. Proper use of the partogram is an essential procedure in midwifery and also helps in clinical decision-making during labour. To this end, the purpose of this study was to describe factors affecting use of the partogram among nurses and midwives in the labour wards of selected public health facilities in the Eastern Province of Rwanda.

In sum, a significant percentage of nurses and midwives in the selected health facilities have fair knowledge of the partogram and why it is necessary to use it in the management of labour. However, despite this, a large percentage of participants reported poor use of the partogram. Nurses’ and midwives’ years of professional experience as well as having received training on managing pregnant women in labour were both found to be predictors of proper use of the partogram.

In addition to these two predictors, the numbers of staff members per shift as well as their professional qualifications demonstrate a significant influence on the proper use of the partogram.

The findings of this study could be useful in designing professional continuing education programmes for nurses and midwives, as well as in formulating policies that may influence effective delivery of care to pregnant women in labour.
